# Supplementary material for: The Rationale for the Dual-Targeting Therapy for RSK2 and AKT in Multiple Myeloma
Source: Int J Mol Sci. 2022 Mar 8;23(6):2919. doi: 10.3390/ijms23062919 (PMC8949999; doi:10.3390/ijms23062919)
Supplement: Supplementary file 1 [file ijms-23-02919-s001.zip › Supplementary information IJMS1590353R2.pdf]

Supplementary information

Supplemental methods

Information about human myeloma-derived cell lines

NCI-H929, OPM-2, and KMS-28-PE cells are positive for t(4;14) translocation which causes MMSET to be deregulated, while KMS-12-BM cells are positive for t(11;14) which induces cyclin D1 overexpression. AMO-1, KMS-12 BM, and KMS-28-PE cells harbor t(8;14) which deregulates c-MYC expression. All HMCLs examined are positive for chromosome 1q gain. NCI-H929 cells possess NRAS G13D mutation, while RPMI8226 and KMS-28-PE cells possess KRAS G12A mutation. OPM-2 cells harbor FGFR3 K650E mutation [28].

Western blot analysis

After lysing cells for 60 minutes at 4°C in RIPA buffer (50 mM Tris-HCl (pH 8.0), 150 mM NaCl, 1% NP-40, 0.5 % deoxycholic acid, 0.1 % SDS, 1 mM DTT, and 0.5 mM PMSF), the lysate was centrifuged at 12,000 rpm for 10 minutes at 4°C, and the supernatant was collected. The protein extract was loaded onto a 12% or 16% SDS-polyacrylamide gel for electrophoresis (Thermo Fisher, Waltham, MA) and transferred to Immobilon-P membranes (Millipore, Burlington, MA). The membranes were blocked in Tris-buffered saline containing 5% skim milk before being incubated with primary antibodies overnight at 4°C. The blots

were then incubated with HRP-conjugated secondary antibody (GE Healthcare. Chicago, IL) for 1 hour at room temperature, and signals were detected using Chemiluminescent HRP Substrate (Millipore) by using ChemiDoc Touch (Bio-Rad Laboratories, Hercules, CA). Primary antibodies used were rabbit monoclonal antibodies against AKT (#4691), BIM (#2933), cleaved Caspase-3 (#9662), cleaved Caspase-8 (#9496), DR5 (#8074), mTOR (#2972), phospho (p)-PDPK1^{Ser241} (#3438), p-PRAS40^{Thr246} (#2997), PRAS40 (#2691), p-RSK2^{Ser227} (#3556), p-mTOR^{Ser2448} (#2971) (Cell Signaling Technology, Danvers, MA); rabbit polyclonal antibodies for BID (#2002), Caspase-9 (#9502), ERK1/2 (#9142), FLIP (#3210), p-AKT^{Ser473} (#9271), p-ERK1/2$^{Thr202/Thr204}$ (#9101), PARP (#9542), PDPK1 (#3062) (Cell Signaling Technology), DR4 (1139) (ProSci, Poway, CA); mouse monoclonal antibodies for β-actin (ACTB) (A5441) (Sigma), Caspase-8 (#9746) (Cell Signaling Technology), c-MYC (sc-40), TNFR1 (sc-8436), RSK2 (sc-9986) (Santa Cruz Biotechnology, Dallas, TX), Fas (SY-001) (Medical & Biological Laboratories, Nagoya, Japan), and TRAIL (NB100-56518) (Novus Biologicals, Centennial, CO).

Lentiviral preparation and transfection

BID and BIM were silenced by the stable expression of inhibitory short hairpin RNAs (shRNAs). The target sequences for human BID were 5′-CTTTCACACAACAGTGAATTT-3′

(shBID#1), 5′-GGTGTTTGGCTTCCTCCAAAG-3′ (shBID#2) and 5′-GGGAAGAATAGAGGCAGATTC-3′ (shBID#3), for human BIM were 5′-TGATGTAAGTTCTGAGTGTG-3′ (shBIM#1) and 5′-GACCGAGAAGGTAGACAATTGC-3′ (shBIM#2), and for negative control was 5′-CCTAAGGTTAAGTCGCCCTCG-3′ (Scramble). The shRNA sequences were cloned into the puro lentivirus vector pLKO.1 (Addgene, Watertown, MA). Transient co-transfection of HEK293T cells with a lentivirus vector, packaging plasmids pMLDg/pRRE, PRSV-Rev, and pMD2.G using PEI-max resulted in the production of lentivirus (Polysciences, Warrington, PA). The viral supernatant was collected 48 hours and 72 hours after transfection and concentrated by ultracentrifugation. Infected cells were selected with 1.0 μg/mL puromycin beginning 48 hours after transfection and maintained under selection for 14 days.

Supplementary Legend

Table S1 **The dose-reduction index (DRI) with BI-D1870, ipatasertib in four RSK2-NTKD- and AKT-activated human myeloma-derived cell lines (HMCLs).** DRI=1, DRI>1, and DRI<1 indicate no dose reduction, favorable, and unfavorable dose-reduction, respectively.

Table S2 **Result of gene expression microarray analysis.**

Table S3 **Primer sequences for quantitative RT-PCR.**

Figure S1 **Original Western blots performed in this study.** Original blots in Figure 1A (**A**), Figure 1D (**B**), Figure 3B (**C**), Figure 3C (**D**), Figure 6 (**E**), Figures S2 (**F**), and Figure S3 (**G**) are shown. Triangles indicate the molecules of interest. Measured values of each band analyzed by Image-J software are described in (A), while the relative expressison levels to controls were described in other figures.

Figure S2 **Effect of knockdown of BIM (A and C) or BID (B and D) in apoptosis by BI-D1870, ipatasertib, or their combination. A and B.** WB for BIM (**A**) and BID (**B**) in parental (Cont.) and gene knockdown (sh.) NCI-H929 cells. SC. Scramble vector. **C and D.** The ratios of apoptosis cells treated by BI-D1870, ipatasertib, or their combination at the indicated concentrations for 48 hours. Ns. No statistical significance was noticed.

Figure S3 **Western blots for DR4, DR5, FAS, TNFR1, FLIP, and TRAIL.** Cells were seeded at $2×10^5$ cells/mL and treated with indicated concentrations of BI-D1870, ipatasertib, or both agents for 48 hours.

Figure S4 **Results of quantitative RT-PCR in NCI-H929 and OPM-2 cells.** NCI-H929 and OPM-2 cells were treated with ipatasertib and/or BI-D1870 at

the indicated concentrations for 24 hours. According to microarray analysis, BI-D1870 significantly increase JUND expression, and the addition of ipatasertib onto BI-D1870 further significantly upregulated JUND expression in both cell lines. Both ipatasertib and BI-D1870 significantly upregulated expression levels of YPEL3, APAF-1, and CDKN1B, the combination of BI-D1870 and ipatasertib further upregulated the expression levels of these three genes. *$p < 0.05$, **$p < 0.01$ ***, $p < 0.001$.
