# Supplementary material for: The Rationale for the Dual-Targeting Therapy for RSK2 and AKT in Multiple Myeloma
Source: Int J Mol Sci. 2022 Mar 8;23(6):2919. doi: 10.3390/ijms23062919 (PMC8949999; doi:10.3390/ijms23062919)
Supplement: Supplementary file 1 [file ijms-23-02919-s001.zip › Table S1 1590353R2.pdf]

Table S1. The dose-reduction index (DRI) with BI-D1870, ipatasertib in four RSK2-NTKD- and AKT-activated human myeloma-derived cell lines (HMCLs).

HMCL	Fraction affected	DRI	
		BI-D1870	ipatasertib
NCI-H929	0.787	3.70	4.01
	0.911	2.47	9.48
	0.967	1.67	7.65
OPM-2	0.642	1.84	13.69
	0.788	2.54	3.39
	0.913	1.34	1.34
KMS-12-BM	0.730	2.53	2.06
	0.827	14.67	1.28
	0.908	3.00	1.68
KMS-28-PE	0.359	2.11	2.05
	0.578	2.563	2.01
	0.801	1.63	2.81
