# Supplementary material for: The Rationale for the Dual-Targeting Therapy for RSK2 and AKT in Multiple Myeloma
Source: Int J Mol Sci. 2022 Mar 8;23(6):2919. doi: 10.3390/ijms23062919 (PMC8949999; doi:10.3390/ijms23062919)
Supplement: Supplementary file 1 [file ijms-23-02919-s001.zip › Table S3 1590353R2.pdf]

Table S3. Primer sequences for quantitative RT-PCR

Gene	Primer sequences
ACTB	Forward: GTCTTCCCCTCCATCGTG<br>Reverse: AGGTGTGGTGCCAGATTTTC
APAF-1	Forward: AAGCTAAGCTGCAGGCCAA<br>Reverse: AACAGCATCTGTGTGGGGG
CDKN1B	Forward: GGCCTGCAGGAACCTCTTC<br>Reverse: GCCCTTCTCCACCTCTTGC
JUND	Forward: ATCGACATGGACACGCAGGAG<br>Reverse: TTCTCTTCCAGGCGCGAGATG
YPEL3	Forward: GACCCTCAACCTTCACGGC<br>Reverse: GGTTGGTCCTTGCGACCTC
