# Supplementary figures and images for: The Rationale for the Dual-Targeting Therapy for RSK2 and AKT in Multiple Myeloma
Source: Int J Mol Sci. 2022 Mar 8;23(6):2919. doi: 10.3390/ijms23062919 (PMC8949999; doi:10.3390/ijms23062919)

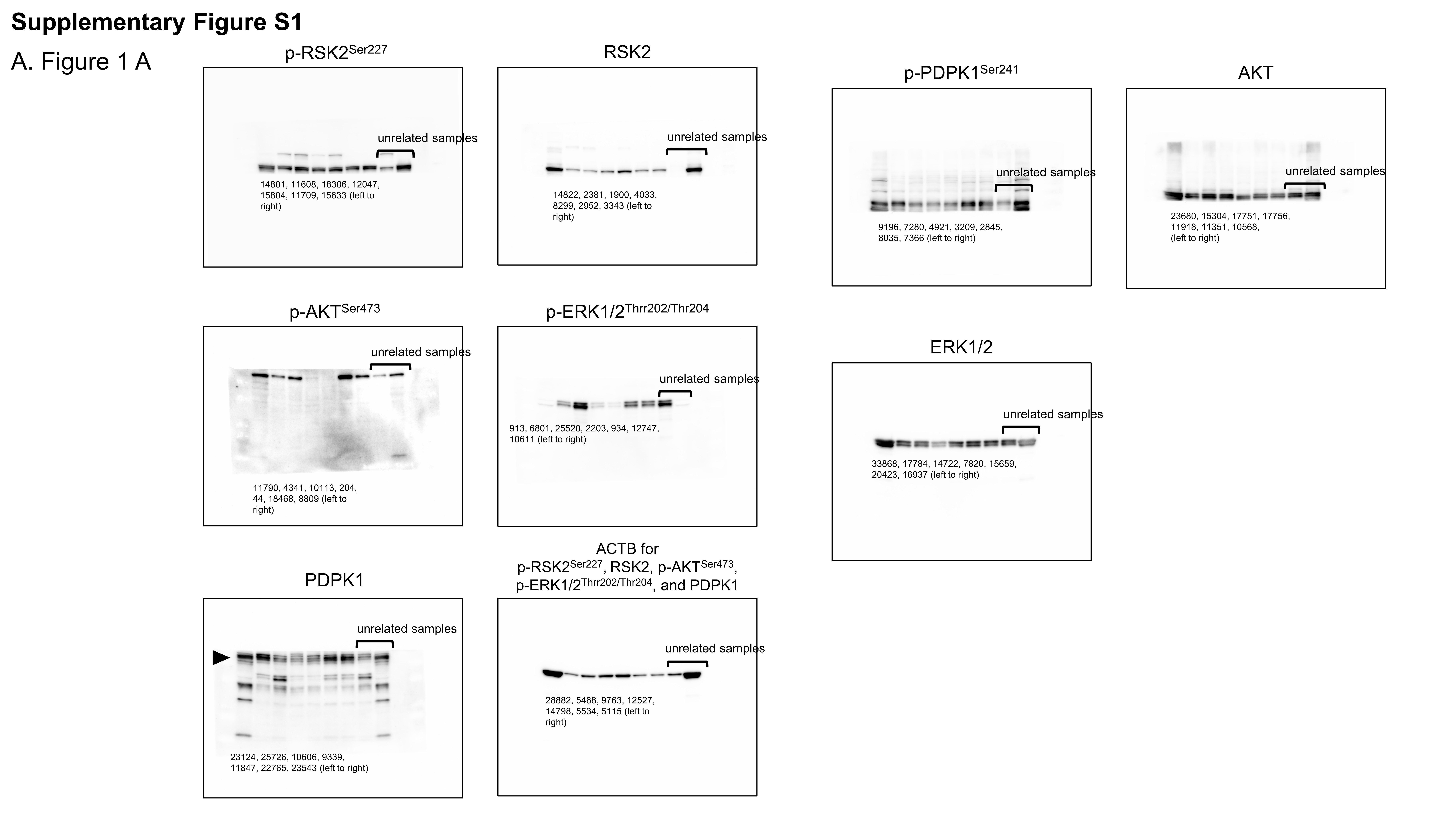

Supplement: Supplementary file 1 [file ijms-23-02919-s001.zip › Figure S1A.JPG]

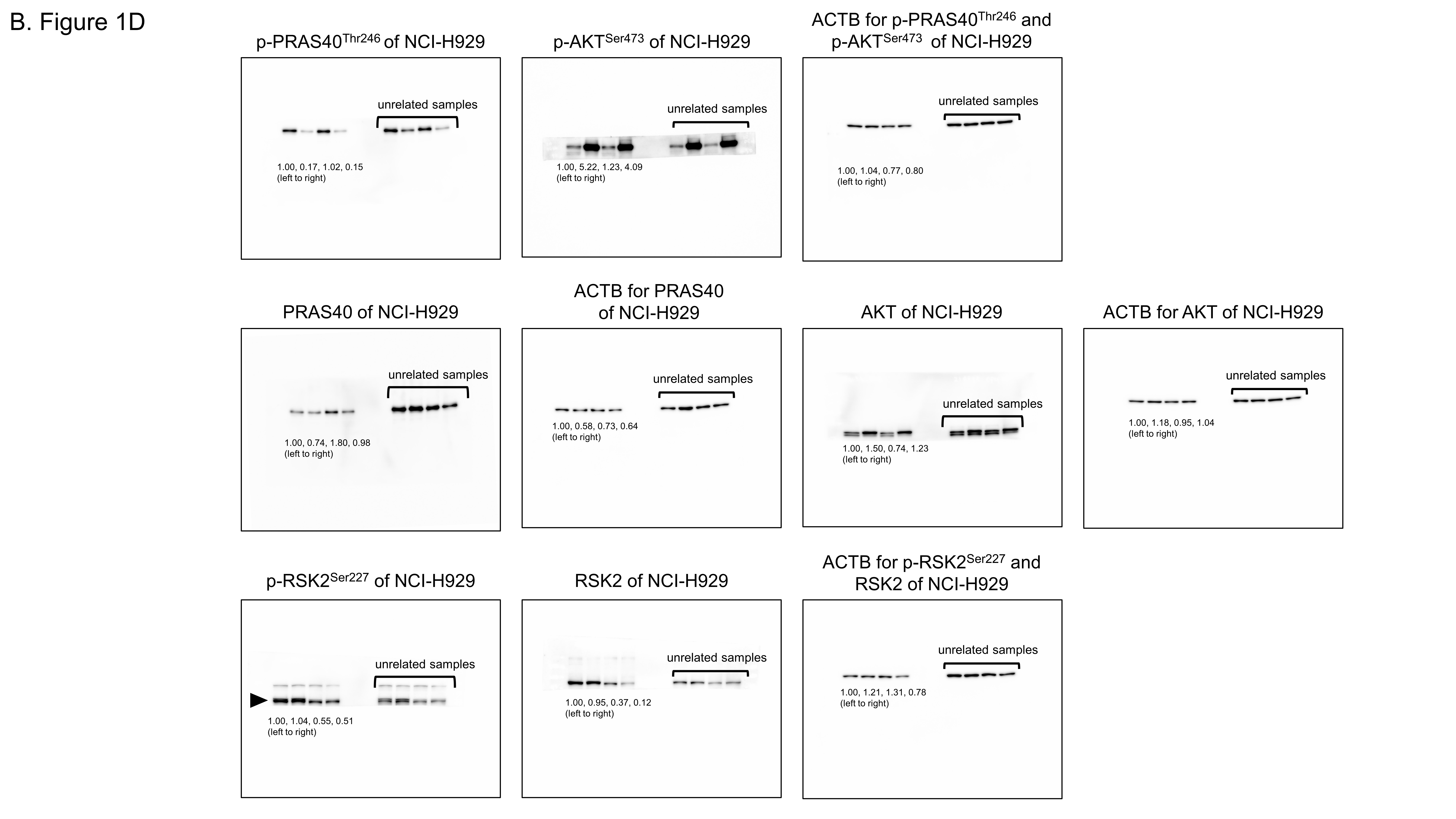

Supplement: Supplementary file 1 [file ijms-23-02919-s001.zip › Figure S1B-1.JPG]

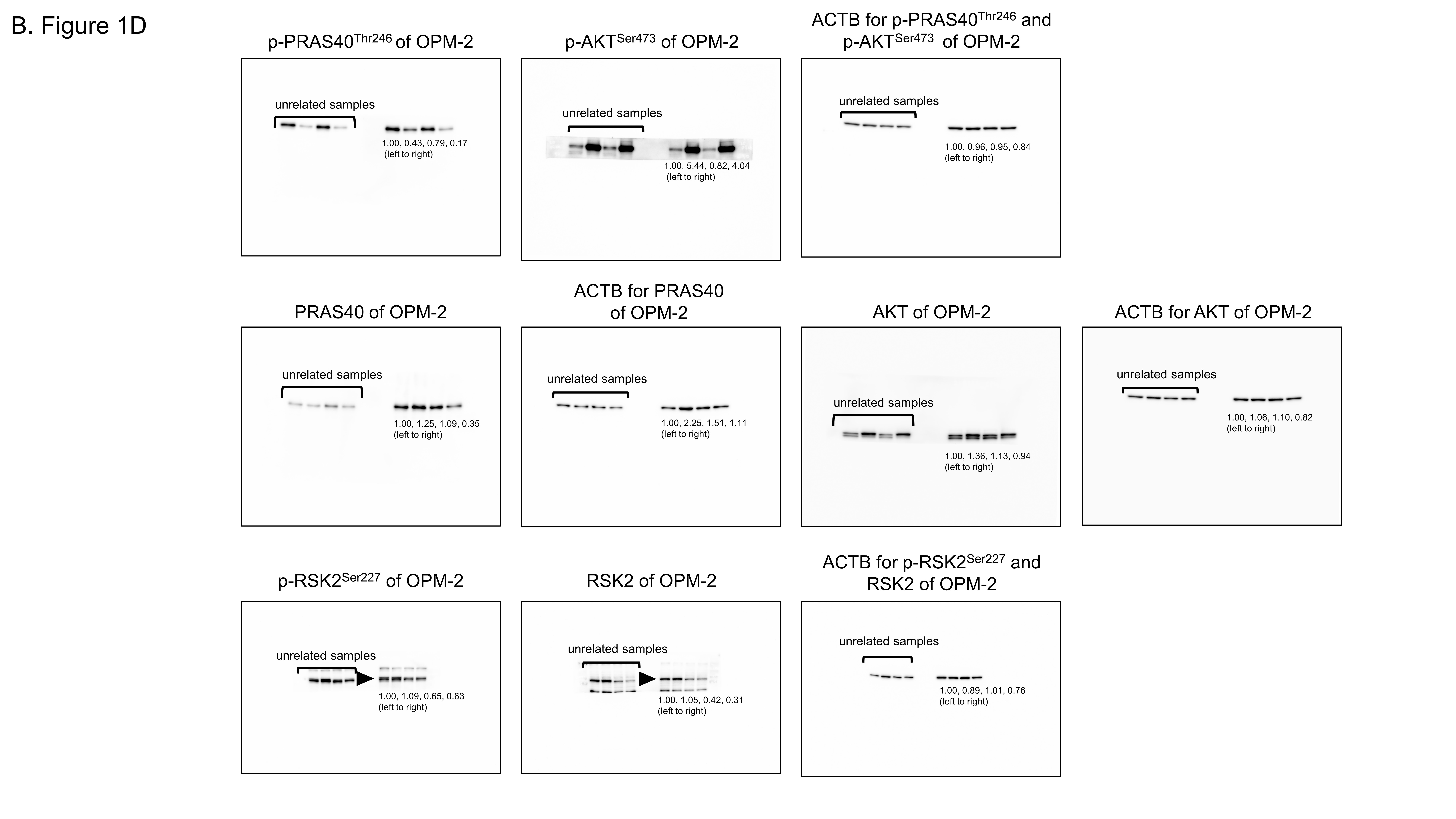

Supplement: Supplementary file 1 [file ijms-23-02919-s001.zip › Figure S1B-2.JPG]

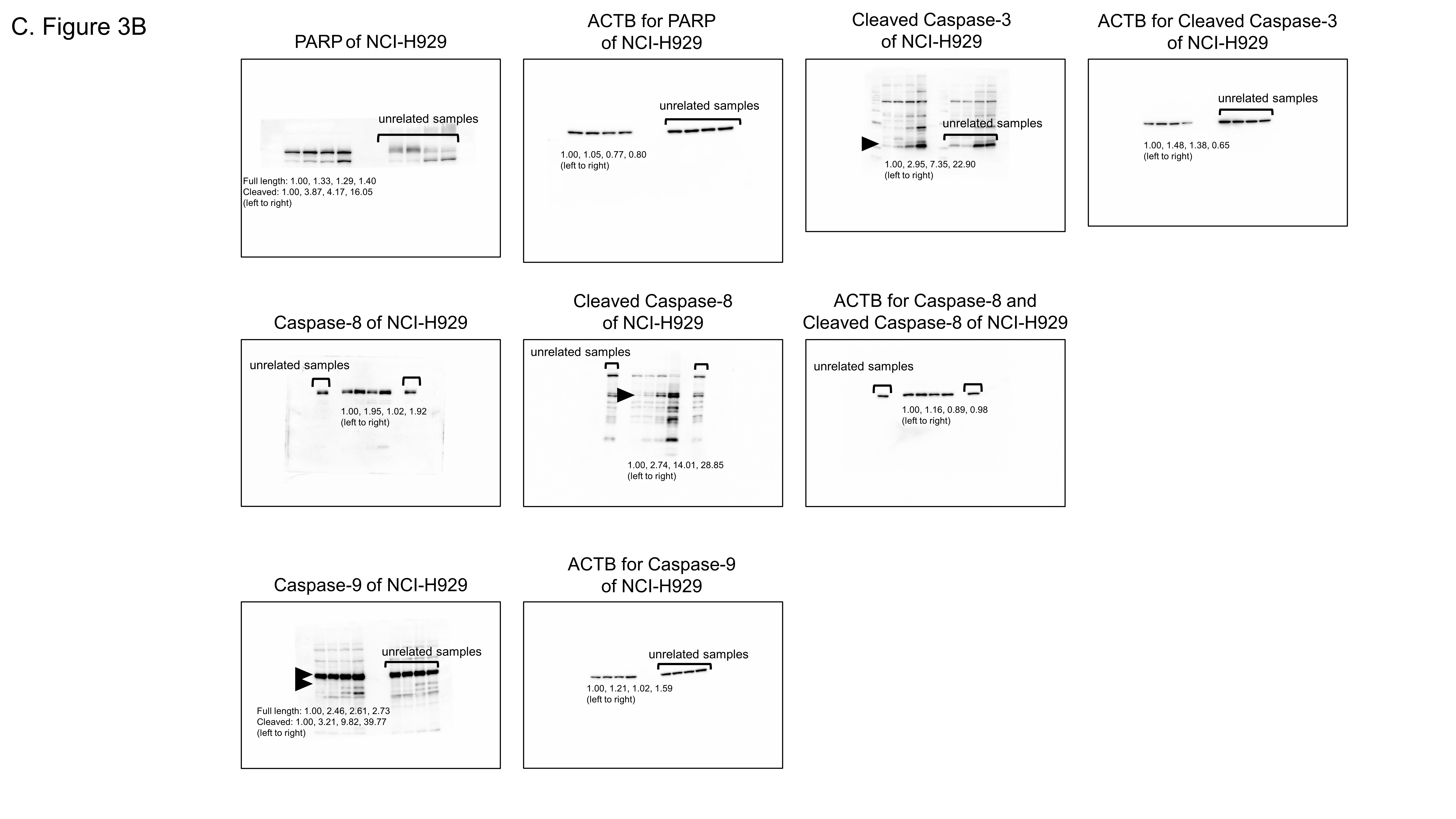

Supplement: Supplementary file 1 [file ijms-23-02919-s001.zip › Figure S1C-1.JPG]

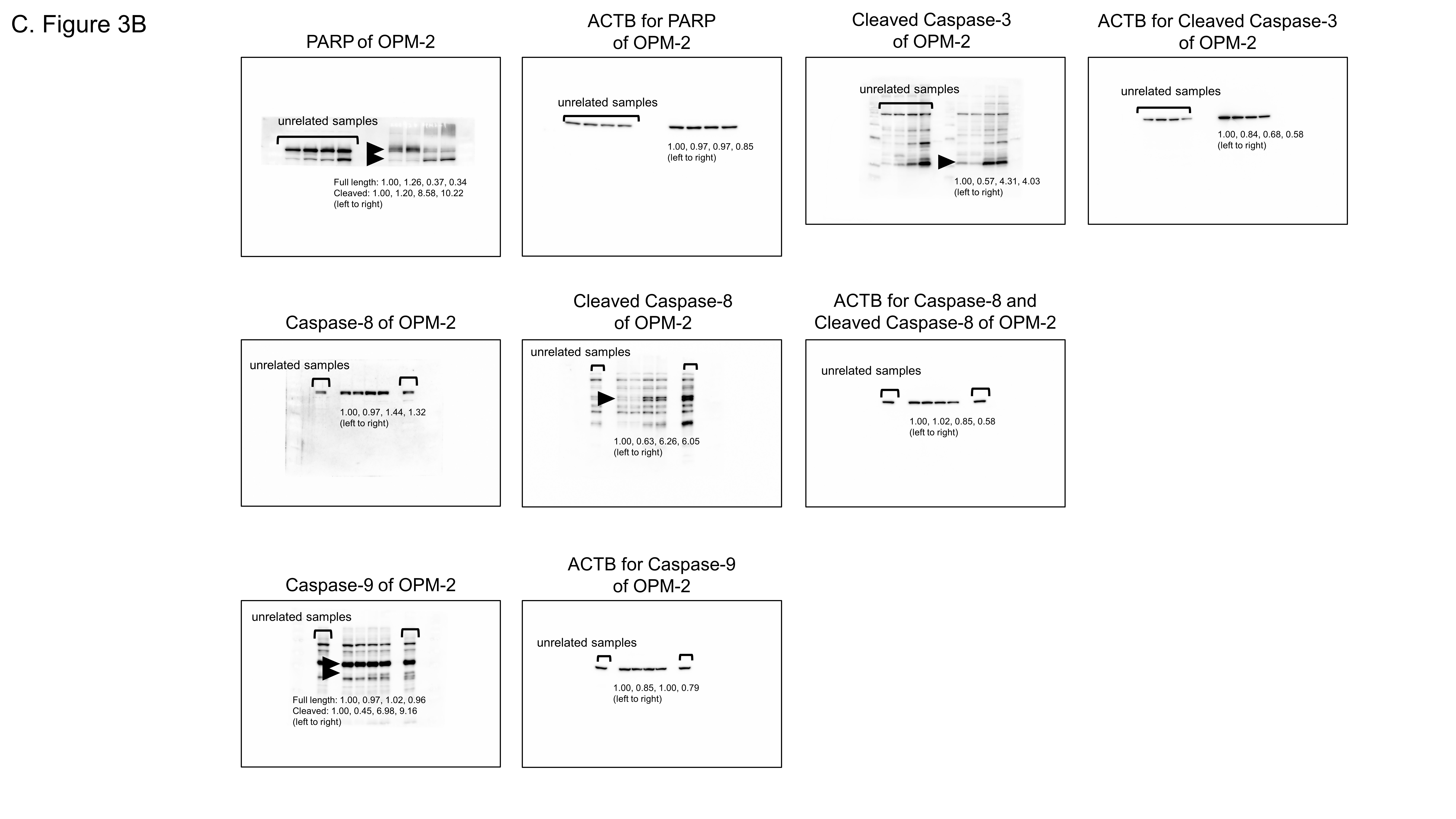

Supplement: Supplementary file 1 [file ijms-23-02919-s001.zip › Figure S1C-2.JPG]

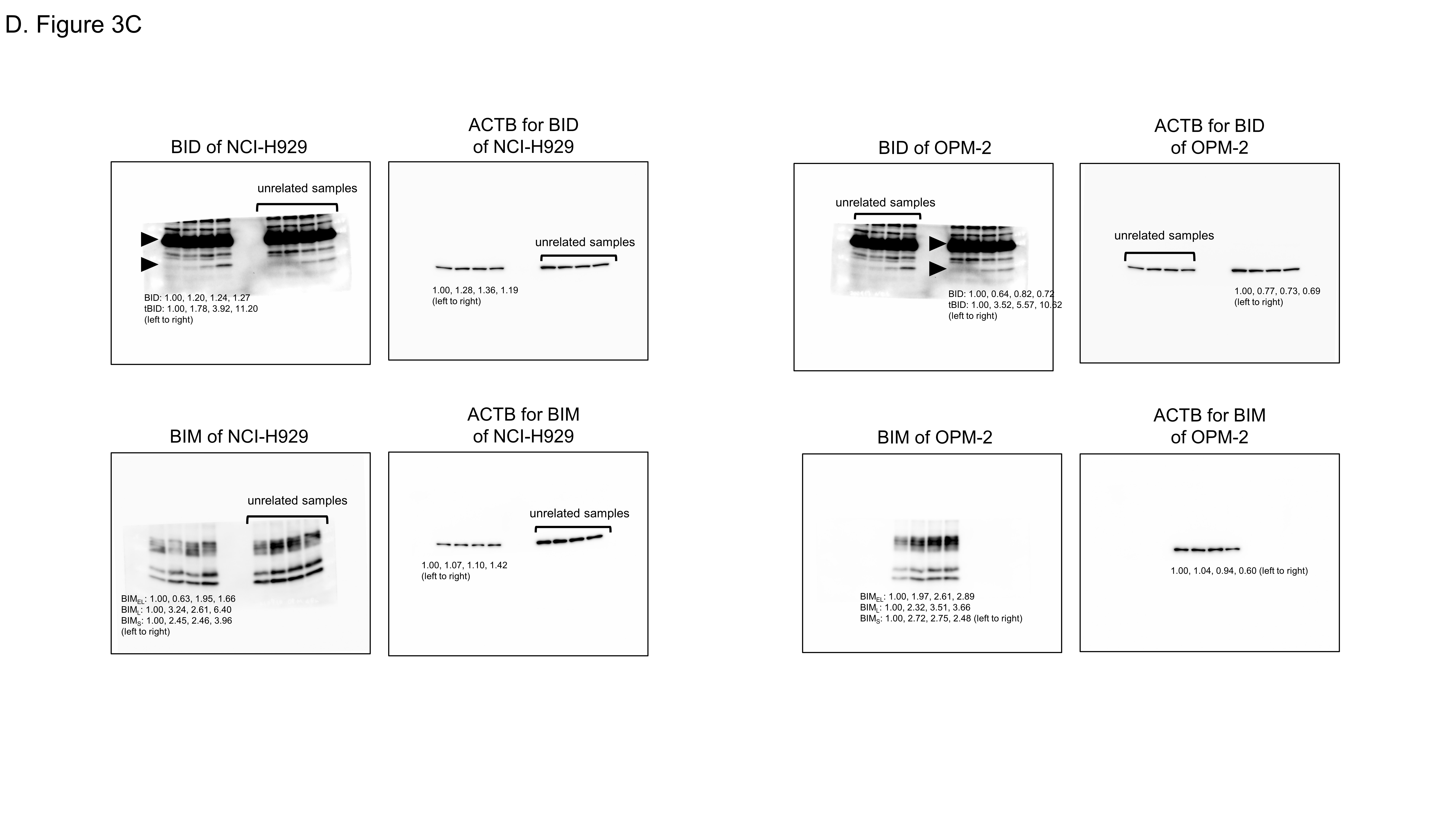

Supplement: Supplementary file 1 [file ijms-23-02919-s001.zip › Figure S1D.JPG]

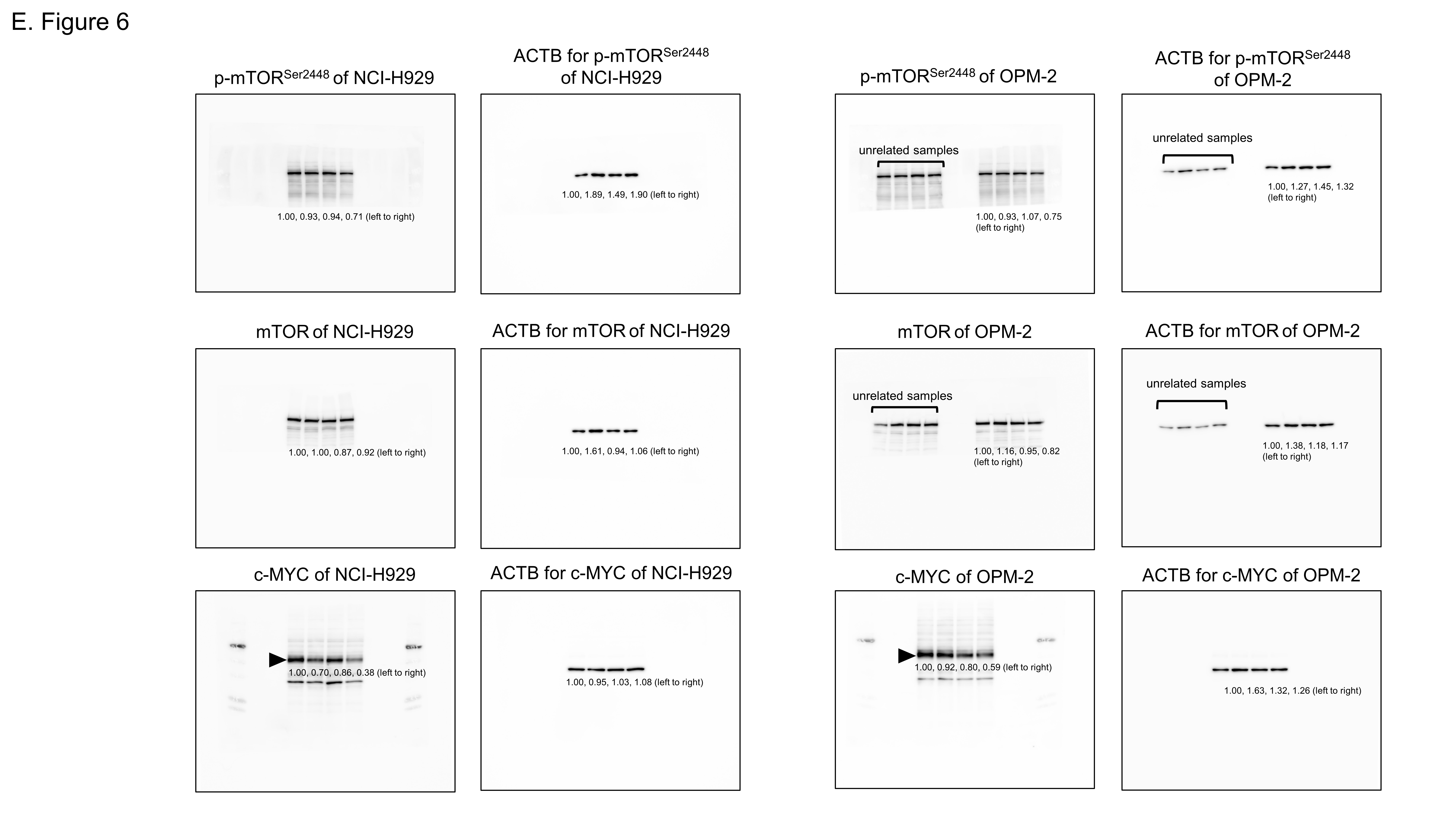

Supplement: Supplementary file 1 [file ijms-23-02919-s001.zip › Figure S1E.JPG]

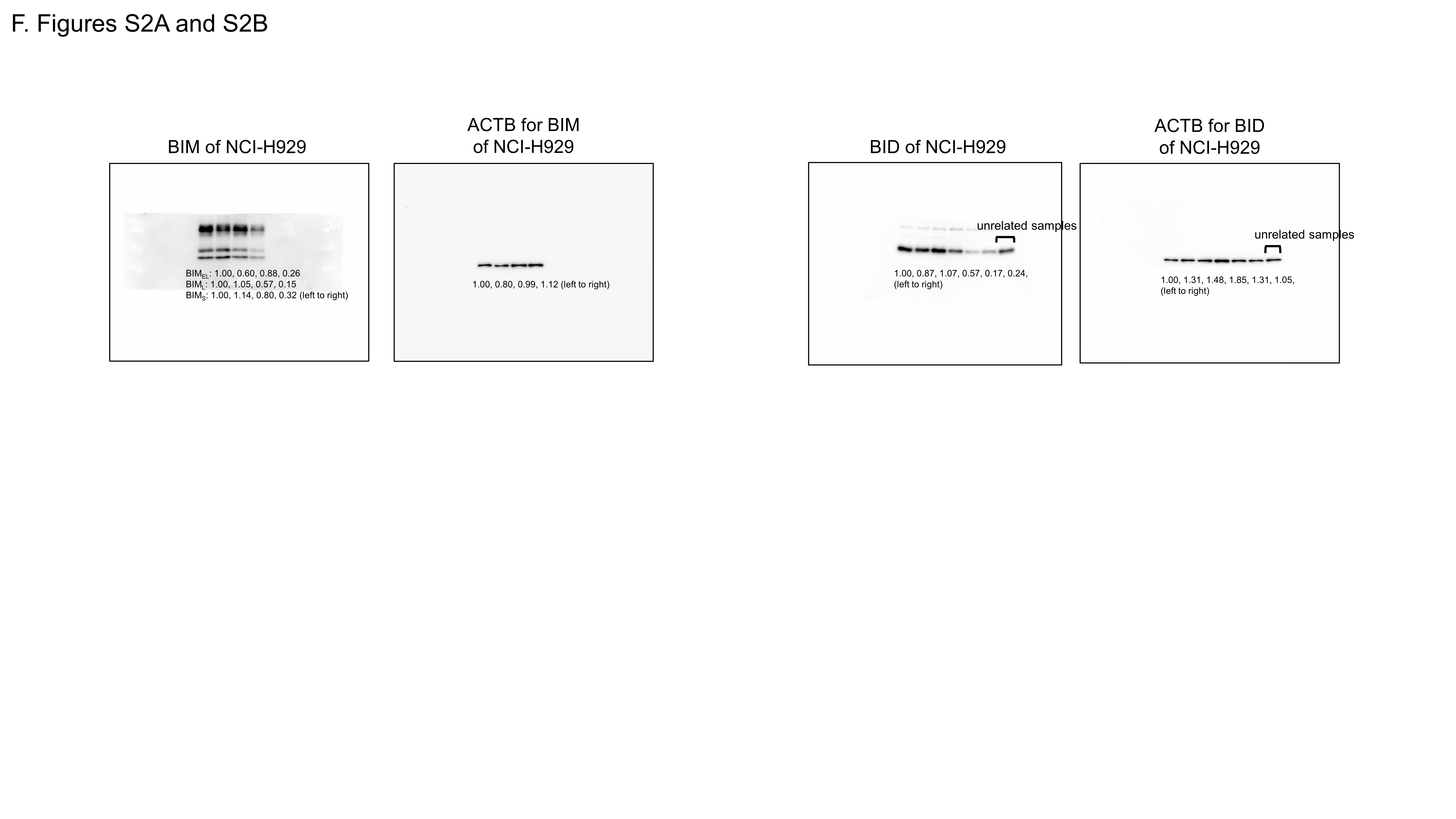

Supplement: Supplementary file 1 [file ijms-23-02919-s001.zip › Figure S1F.JPG]

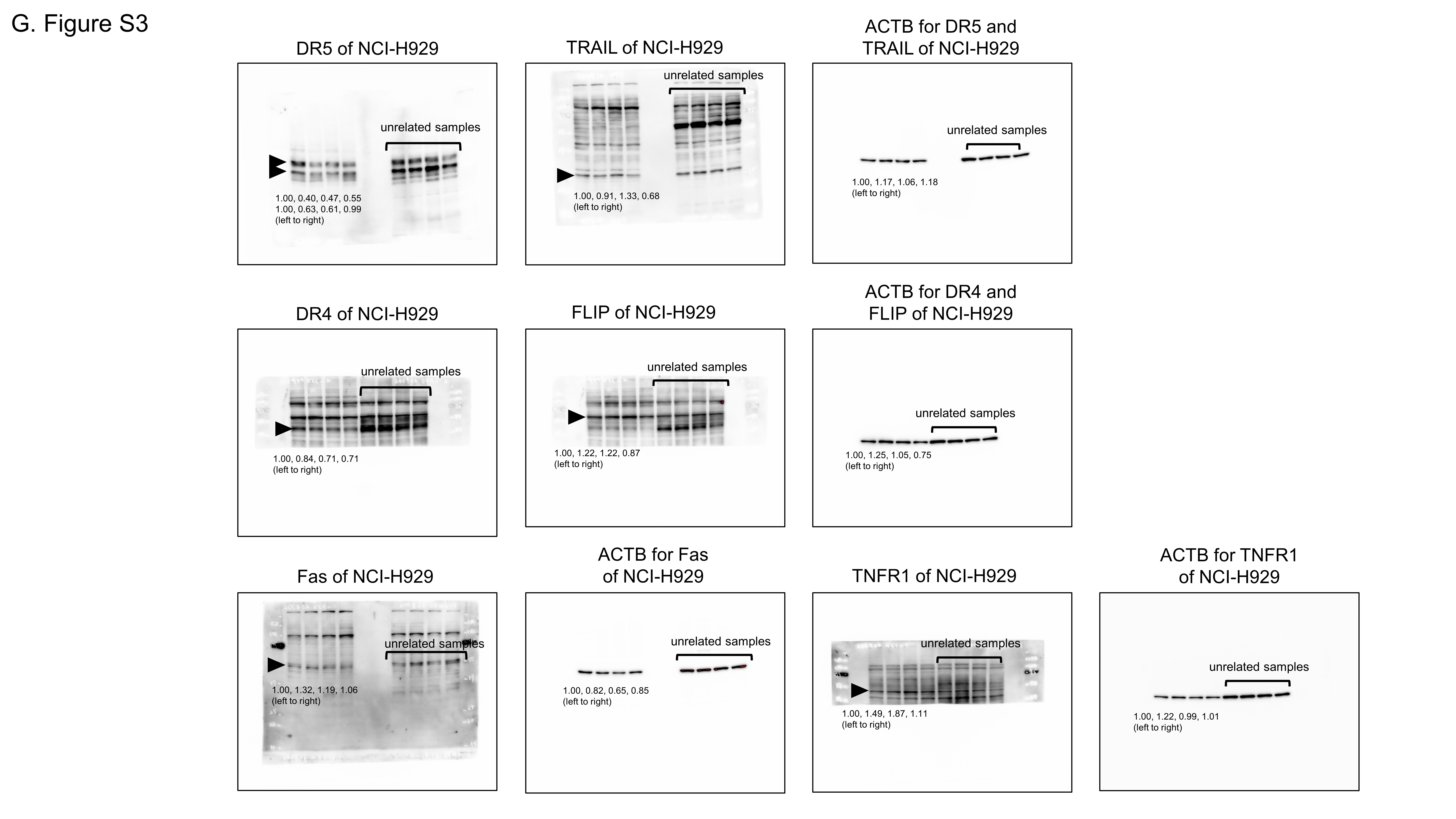

Supplement: Supplementary file 1 [file ijms-23-02919-s001.zip › Figure S1G-1.JPG]

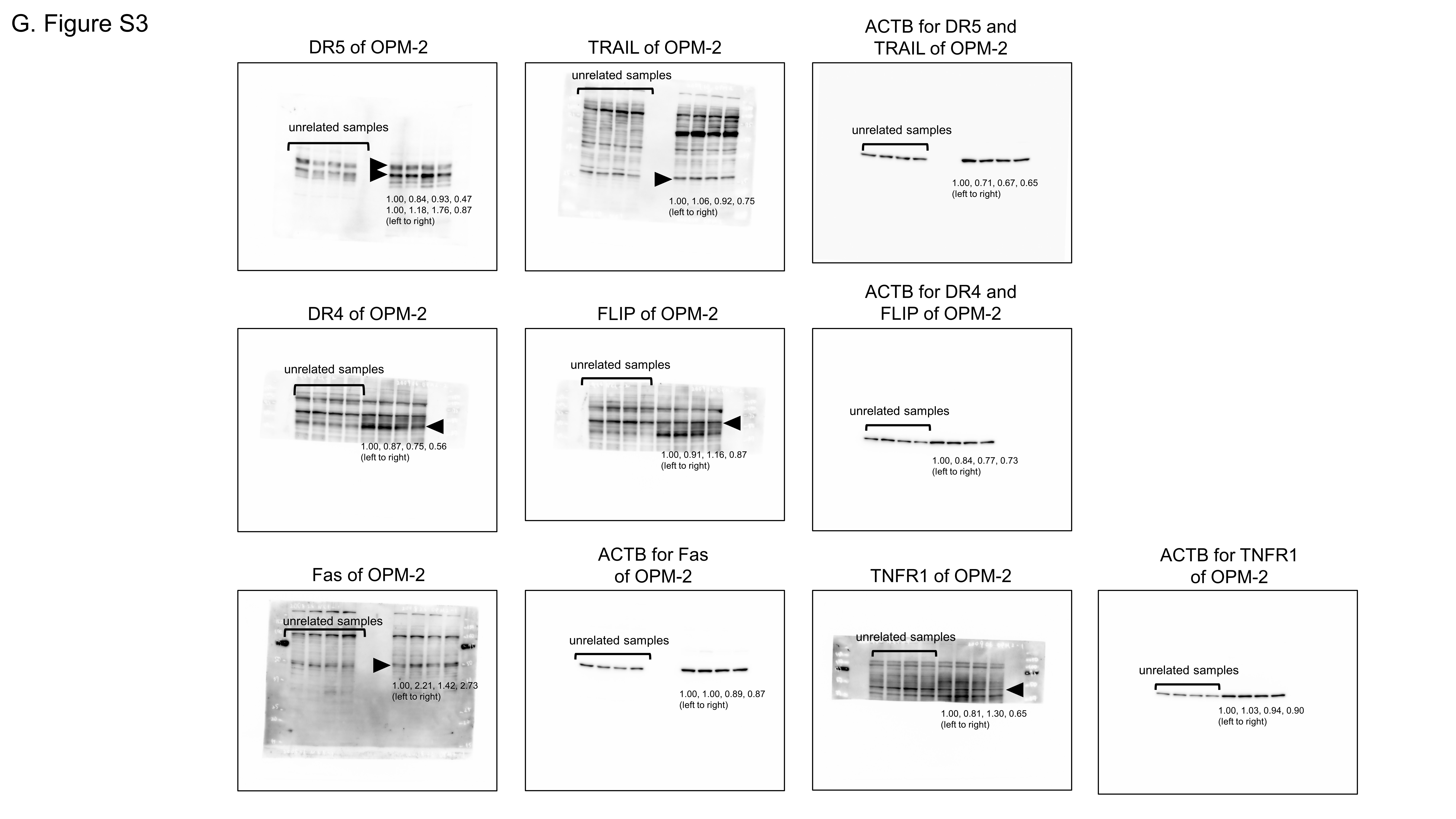

Supplement: Supplementary file 1 [file ijms-23-02919-s001.zip › Figure S1G-2.JPG]

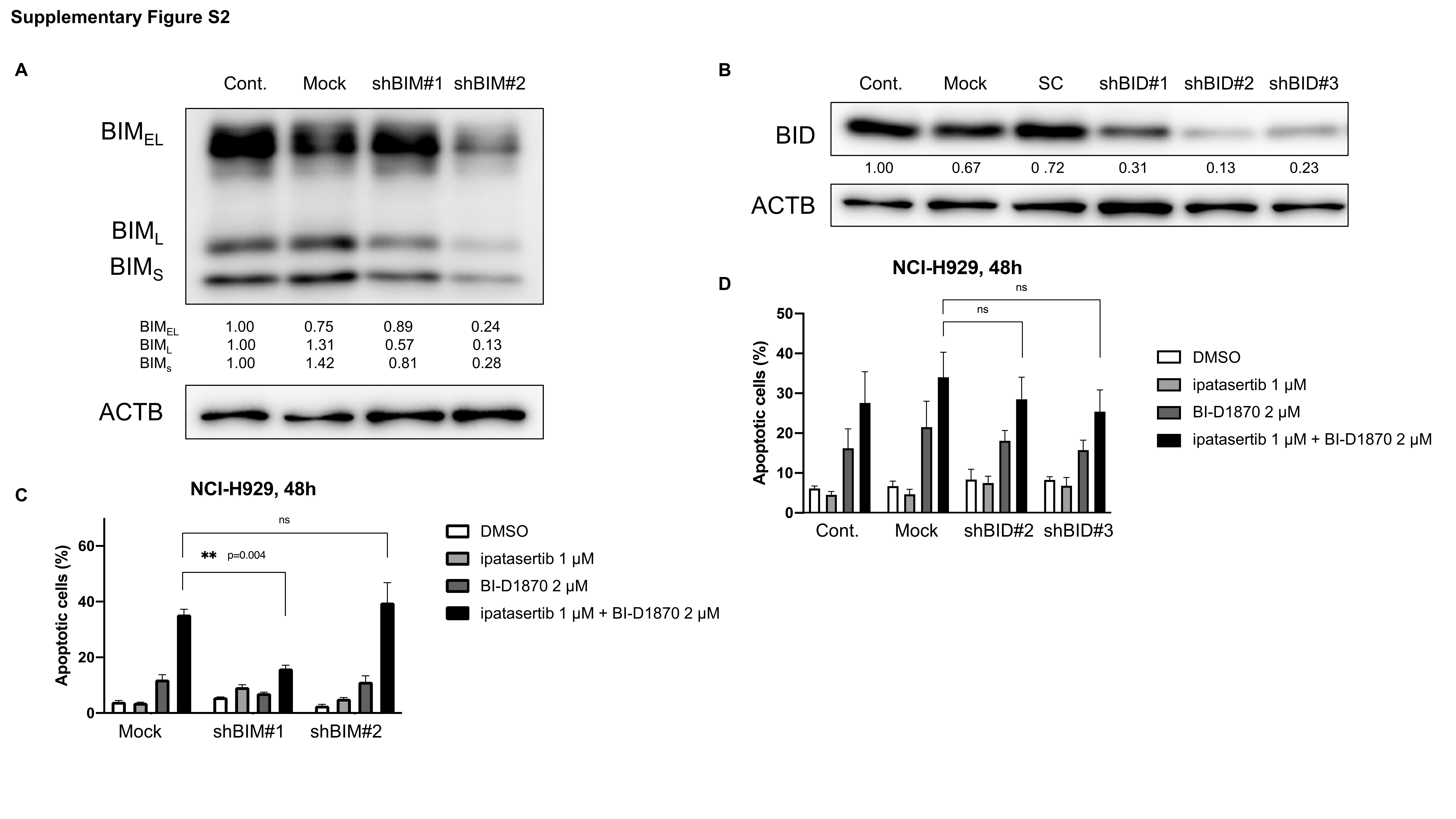

Supplement: Supplementary file 1 [file ijms-23-02919-s001.zip › Figure S2R1.jpg]

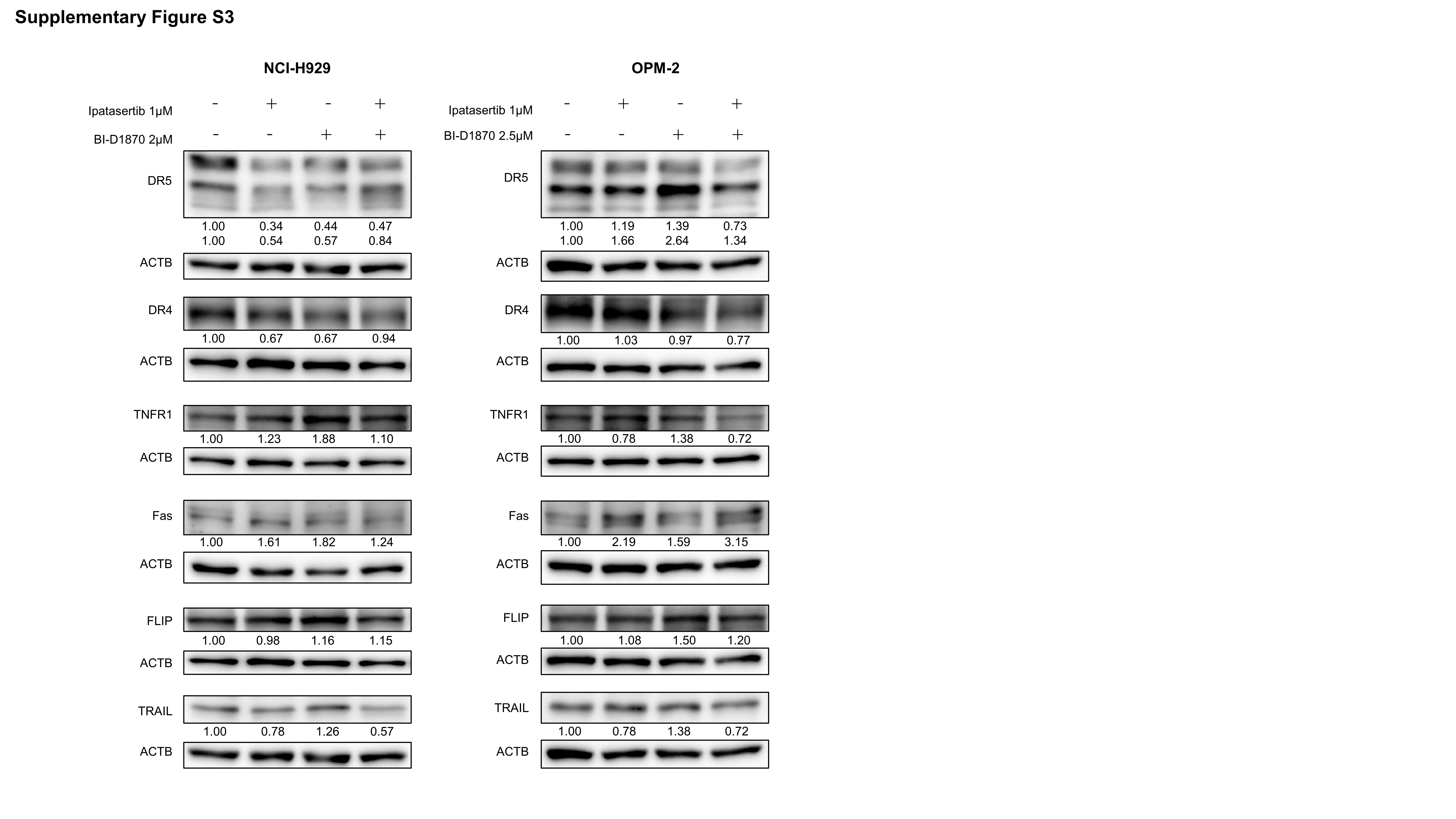

Supplement: Supplementary file 1 [file ijms-23-02919-s001.zip › Figure S3R1.jpg]

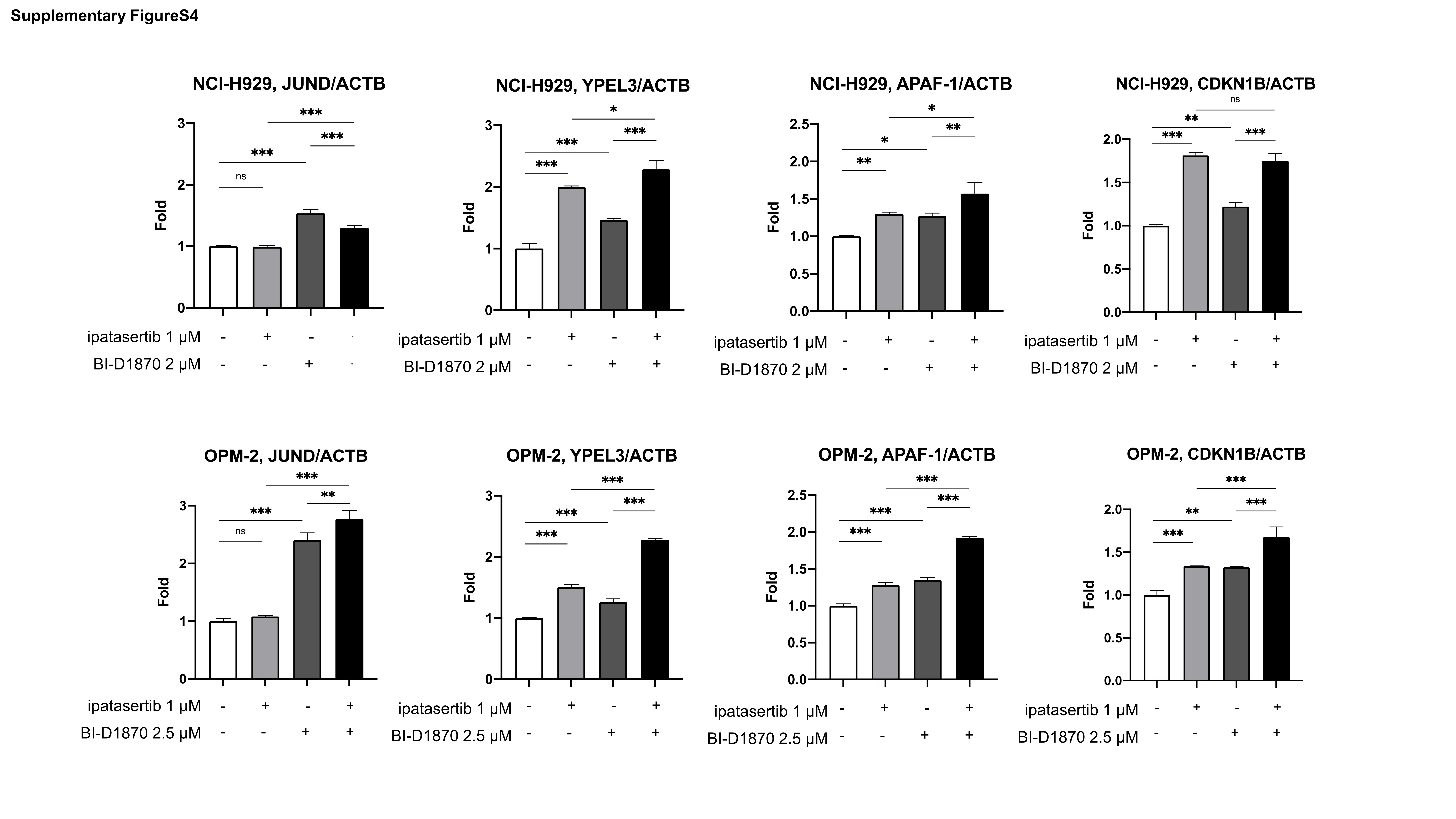

Supplement: Supplementary file 1 [file ijms-23-02919-s001.zip › Figure S4R1.jpg]
